# Early risk stratification for carbapenem resistance among *Pseudomonas aeruginosa* infected patients using a clinico-laboratory machine-learning model based on routine complete blood count parameters

**DOI:** 10.3389/fcimb.2026.1795720

**Published:** 2026-04-16

**Authors:** Jianhong Yu, Wei Gao, Yu Chen

**Affiliations:** 1 Department of Clinical Laboratory, Zigong First People’s Hospital, Zigong, China; 2 Department of Clinical Laboratory, Ya’an People’s Hospital, Ya’an, China

**Keywords:** carbapenem-resistant *Pseudomonas aeruginosa*, clinico-laboratory omic, complete blood count, machine learning, predictive model, risk stratification

## Abstract

**Introduction:**

To address the delayed identification of carbapenem-resistant *Pseudomonas aeruginosa* (CRPA), we developed an interpretable machine-learning (ML) model for early risk stratification. Utilizing routine complete blood count (CBC) and demographic data, this tool targets the critical 48–72 hour interval before final antimicrobial susceptibility results.

**Methods:**

Data from 1,666 patients with *P. aeruginosa* infection (223 CRPA) at a primary center were retrospectively analyzed, alongside an independent external validation cohort (n=471). Following the least absolute shrinkage and selection operator (LASSO) regression on 32 variables, eight ML algorithms were trained. Model interpretability and clinical utility were evaluated using Shapley Additive Explanations (SHAP) and decision curve analysis (DCA). Eight ML algorithms were trained using 5-fold cross-validation and Bayesian hyperparameter optimization. To ensure reproducibility and handle class imbalance, fixed random seeds were set, and a sensitivity analysis using the Synthetic Minority Over-sampling Technique (SMOTE) was conducted. Model calibration was assessed using the Brier score.

**Results:**

LASSO identified seven predictors: sex, age, mean corpuscular volume (MCV), hemoglobin (HGB), platelet-to-lymphocyte ratio (PLR), systemic inflammatory response index (SIRI), and intensive care unit (ICU) admission status. Among the evaluated algorithms, the random forest (RF) model achieved the best discrimination. The training area under the receiver operating characteristic curve (AUC) was 0.993; it achieved an average 5-fold cross-validation AUC of 0.929 ± 0.005. In the internal test set, it achieved an AUC of 0.837 (95% CI: 0.779–0.893), specificity of 0.972, and sensitivity of 0.507, with excellent calibration (Brier score = 0.084). The model retained strong performance externally (AUC: 0.898, specificity: 0.985, sensitivity: 0.600, Brier score: 0.073). SHAP analysis indicated that HGB was the most influential feature, inversely associated with CRPA risk. Decision curve analysis supported the clinical utility across threshold probabilities ranging from 15% to 65%.

**Discussion:**

This clinlabomics-based RF model provides a rapid, low-cost adjunct for early CRPA stratification. Given its exceptionally high specificity (>0.97) and modest sensitivity, it functions exclusively as a reliable clinical “rule-in” tool. Positive predictions can confidently guide early targeted therapy and strict infection control. However, negative predictions cannot safely rule out CRPA, emphasizing its role alongside standard empirical practices rather than as a standalone screening instrument.

## 1 Introduction

The escalating global burden of antimicrobial resistance (AMR) has profoundly impacted the clinical management of infectious diseases, posing a persistent public health challenge and incurring substantial socioeconomic costs (GBD 2021 Antimicrobial Resistance Collaborators, 2024). Among multidrug-resistant pathogens, *Pseudomonas aeruginosa*—a versatile Gram-negative opportunistic bacterium—represents a major therapeutic challenge. Designated a “High Priority” pathogen on the WHO 2024 Bacterial Priority Pathogens List (BPPL), carbapenem-resistant *P. aeruginosa* (CRPA) exhibits considerable environmental adaptability and genomic plasticity (Sati et al., 2025). CRPA infections are associated with markedly adverse outcomes; meta-analyses report attributable mortality of approximately 30% in CRPA bloodstream infections, substantially higher than in carbapenem-susceptible cases (Buehrle et al., 2016; Ahmadi et al., 2025). The spread of CRPA, driven by both intrinsic and acquired resistance mechanisms that compromise carbapenem efficacy, remains a critical clinical concern (Muderris et al., 2018).

Conventional detection and management of CRPA rely on culture-based methods and phenotypic susceptibility testing. Although these approaches are considered the diagnostic gold standard, they typically require 48–72 hours from specimen collection to final results. This delay creates a critical therapeutic window in which empirical broad-spectrum antibiotics are frequently initiated, potentially worsening clinical outcomes and intensifying selective pressure for resistance (Tamma et al., 2023). Although rapid molecular diagnostics (e.g., PCR) and MALDI-TOF mass spectrometry have accelerated pathogen identification, these technologies are often costly, require specialized infrastructure, and do not capture dynamic host physiological responses in real time (Rocca et al., 2025). Accordingly, there is a clear need for rapid, accessible tools to stratify carbapenem resistance risk among patients with culture-confirmed *P. aeruginosa* infections during the critical 48–72 hour diagnostic window preceding final antimicrobial susceptibility testing (AST) results.

Within the framework of precision medicine, machine learning (ML) offers a promising strategy for extracting predictive signals from complex clinical datasets (Rajkomar et al., 2019). Although whole-genome sequencing (WGS) may enable resistance prediction, its cost and technical requirements often preclude routine clinical implementation (Kim et al., 2022). In contrast, “clinlabomic” data generated from routine laboratory testing constitute a widely available yet underutilized resource (Wen et al., 2022). Integrating clinico-laboratory omic (clinlabomics) with artificial intelligence (AI) may help overcome existing diagnostic bottlenecks (Steinbach et al., 2024). In particular, complete blood count (CBC) parameters provide a low-cost, timely reflection of host immune status. Beyond conventional measures, derived indices such as the neutrophil-to-lymphocyte ratio (NLR) and platelet-to-lymphocyte ratio (PLR) have demonstrated prognostic value in bacterial infections and sepsis (Chang et al., 2023; Gunčar et al., 2024). Unlike selective biomarkers such as procalcitonin, CBC testing is performed in nearly all hospitalized patients, providing a consistent and practical basis for predictive modeling (Lien et al., 2022).

Despite increasing interest in ML-based infection prediction, models specifically developed for differentiating CRPA from susceptible strains remain limited, representing an important gap given the consequences of delayed or inappropriate therapy (Zhao et al., 2025). Existing models often rely on numerous variables that may not be consistently available across institutions, thereby constraining generalizability (Song et al., 2020). Furthermore, many high-performing algorithms function as “black boxes”, limiting transparency and impeding clinical trust and uptake (Busnatu et al., 2022). Interpretability is therefore essential for elucidating the potentially non-linear relationships between biomarkers and clinical risk (Kawakami et al., 2021).

To address these limitations, we developed and validated an interpretable ML framework using only routinely available clinlabomic (CBC-derived) and basic demographic data. We systematically compared eight ML algorithms, employed LASSO for robust feature selection, and used SHAP to quantify and visualize predictor contributions. External validation was performed to rigorously assess generalizability. This study aimed to determine whether subtle, integrated patterns within standard CBC parameters could serve as early indicators of carbapenem resistance risk within the critical 48–72 hour diagnostic window—an innovative, clinically pragmatic approach that remains underexplored.

## 2 Patients and methods

### 2.1 Participants

**Figure 1:**
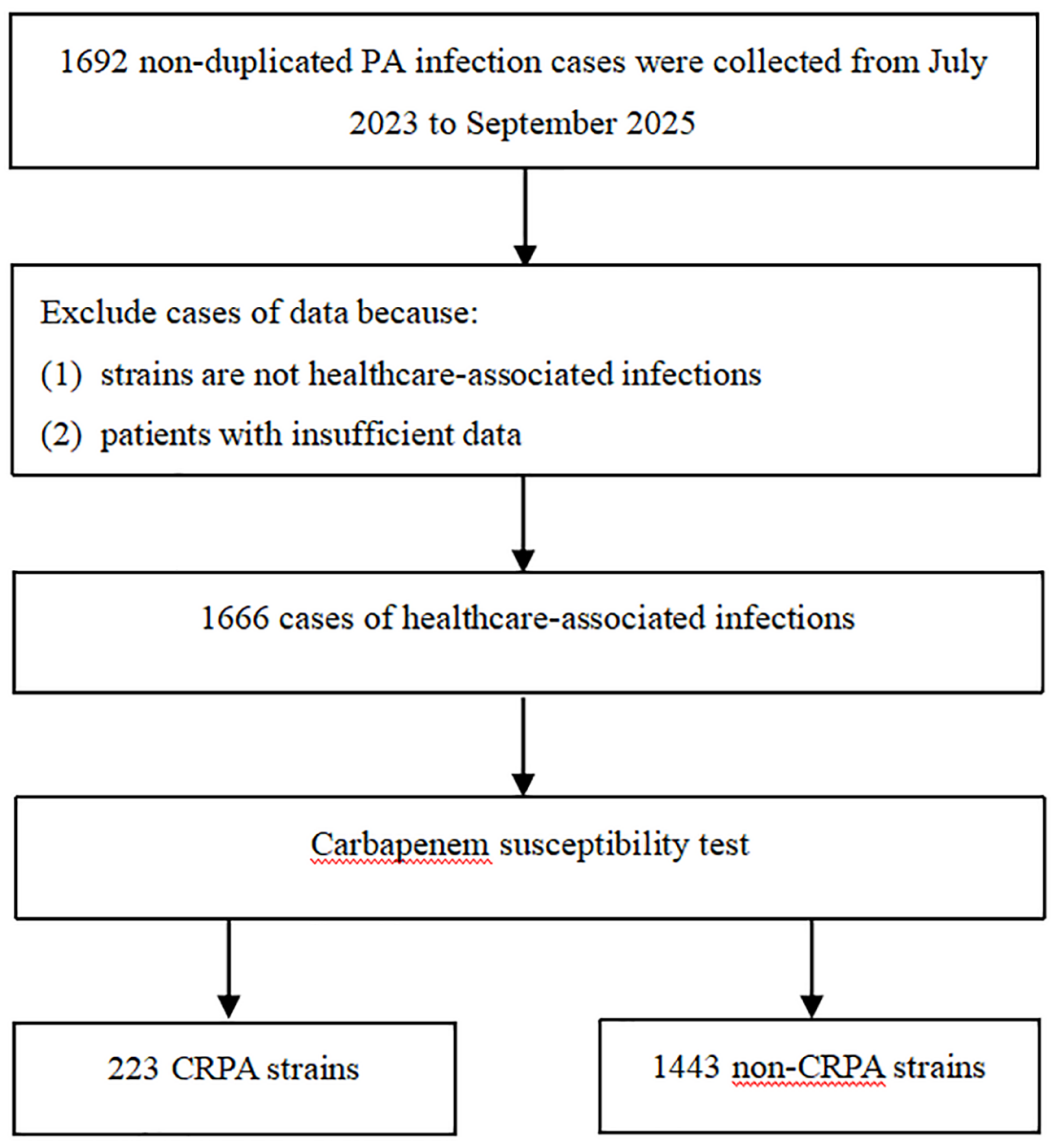
Cases were included in a flow chart. PA, *Pseudomonas aeruginosa*; CRPA, carbapenem-resistant *Pseudomonas aeruginosa*; non-CRPA, non-carbapenem-resistant *Pseudomonas aeruginosa*. (Records with “insufficient” data were rigorously excluded).

This retrospective case–control study was conducted at Zigong First People’s Hospital (a tertiary Grade A hospital) between July 2023 and September 2025. Hospitalized patients with laboratory-confirmed *P. aeruginosa* infection were screened for eligibility. The clinical diagnosis of true infection, as opposed to colonization, was rigorously adjudicated by the hospital’s infection control department and treating clinicians based on the Centers for Disease Control and Prevention/National Healthcare Safety Network (CDC/NHSN) criteria, requiring both positive microbiological cultures and concurrent clinical signs of infection (e.g., fever, purulent exudate, elevated inflammatory markers, or imaging evidence). Inclusion criteria were: (1) age ≥18 years; and (2) availability of complete blood count (CBC) data obtained within 24 hours before the first positive culture. Exclusion criteria were: (1) inactive infection status (e.g., colonization in the absence of clinical symptoms); (2) isolates with an “intermediate” susceptibility phenotype to carbapenems; and (3) insufficient or incomplete clinical or laboratory records (defined as missing any of the targeted demographic or CBC parameters). Consequently, a complete-case analysis approach was utilized, and no missing data imputation techniques were required. After applying these criteria, 1,666 patients were included in the development cohort (**Figure 1**). An independent external validation cohort was established using data from Ya’an People’s Hospital collected between January 2024 and December 2025. This study was approved by the Institutional Ethics Committee of Zigong First People’s Hospital (Approval No. 70, 2024), with a waiver of informed consent granted due to its retrospective design.

### 2.2 Definitions

Carbapenem-resistant *P. aeruginosa* (CRPA): *P. aeruginosa* isolates resistant to either imipenem or meropenem, as determined by antimicrobial susceptibility testing.

Non-CRPA: *P. aeruginosa* isolates susceptible to both imipenem and meropenem.

Complete blood count (CBC) parameters: routine hematological indices obtained from venous blood samples, including cell counts and derived inflammatory ratios.

Derived inflammatory indices: the neutrophil-to-lymphocyte ratio (NLR), platelet-to-lymphocyte ratio (PLR), systemic immune–inflammation index (SII), systemic inflammatory response index (SIRI), lymphocyte-to-monocyte ratio (LMR), and neutrophil-to-platelet ratio (NPR), calculated as described in Section 2.3.

### 2.3 Data collection

Data were extracted from the hospital laboratory information system (LIS). The variables collected were as follows: (1) Demographic characteristics: sex, age, admitting department, and intensive care unit (ICU) admission status, and primary specimen sources (e.g., sputum, abdominal fluid, urine). (2) Complete blood count (CBC) parameters: white blood cell count (WBC), neutrophil count (N), lymphocyte count (L), monocyte count (Mon), eosinophil count (Eo), basophil count (Bas), red blood cell count (RBC), hemoglobin (HGB), hematocrit (HCT), red cell distribution width (RDW-SD and RDW-CV), mean corpuscular volume (MCV), mean corpuscular hemoglobin (MCH), mean corpuscular hemoglobin concentration (MCHC), platelet count (PLT), mean platelet volume (MPV), plateletcrit (PCT), platelet distribution width (PDW), and platelet large cell ratio (PLCR). (3) Inflammatory indices: neutrophil-to-lymphocyte ratio (NLR), platelet-to-lymphocyte ratio (PLR), systemic immune-inflammation index (SII), systemic inflammatory response index (SIRI), lymphocyte-to-monocyte ratio (LMR), and neutrophil-to-platelet ratio (NPR), calculated from the corresponding CBC parameters. (4) Microbiological data: bacterial identification was performed using matrix-assisted laser desorption/ionization time-of-flight mass spectrometry (MALDI-TOF MS). Carbapenem susceptibility was assessed using the VITEK 2 Compact system (bioMérieux, Marcy-l’Étoile, France) for imipenem and the disk diffusion method for meropenem (Wenzhou Kangtai, Zhejiang, China), in accordance with the Clinical and Laboratory Standards Institute (CLSI) guidelines (CLSI, 2025).

### 2.4 Machine-learning model development and validation

All statistical and machine-learning analyses were performed in Python (version 3.9.23) using the scikit-learn framework. To ensure full reproducibility of the models, a fixed global random seed (e.g., random_state = 123) was set for all data splitting, cross-validation, and model training processes. Before model development, data preprocessing was systematically conducted. Given that machine-learning algorithms (particularly LASSO, SVM, and KNN) are highly sensitive to the scale of input features, all continuous variables were standardized using Z-score normalization (StandardScaler), scaling the data to have a mean of 0 and a variance of 1. Categorical variables (e.g., sex, ICU admission status) were encoded as binary indicators. The dataset was randomly partitioned into a training set (70%) and an internal test set (30%) using a stratified splitting technique to preserve the original prevalence ratio of CRPA to non-CRPA cases across both sets. To rule out the possibility of data imbalance skewing the results, the baseline characteristics between the training and internal test datasets were statistically compared, confirming that there were no significant differences across all demographic and laboratory variables (**Supplementary Table 1**). Feature selection was performed using the least absolute shrinkage and selection operator (LASSO) regression to reduce dimensionality and mitigate multicollinearity. To address class imbalance during training, we applied a class weighting strategy inverse to the class frequency. Additionally, a sensitivity analysis utilizing the Synthetic Minority Over-sampling Technique (SMOTE) was performed to evaluate alternative handling of the imbalanced data. Eight machine-learning algorithms were trained and compared: random forest (RF), logistic regression (LG), decision tree (DT), support vector machine (SVM), k-nearest neighbors (KNN), gradient boosting machine (GBM), extreme gradient boosting (XGB), and light gradient boosting machine (LGBM). Hyperparameters were optimized using Bayesian search (**Supplementary Table 2**). To further evaluate the model robustness and address potential overfitting observed in the random forest algorithm, a 5-fold cross-validation was conducted on the training dataset. The distribution of the cross-validated area under the curve (AUC) was calculated and reported to provide a more realistic estimate of the model’s performance on unseen data. The final model was evaluated on the held-out internal test set and the external validation cohort from Ya’an People’s Hospital. Furthermore, model calibration was quantitatively assessed using the Brier score and visually via calibration curves to ensure predicted probabilities aligned with observed frequencies. Model interpretability was assessed using Shapley additive explanations (SHAP) to visualize feature contributions.

### 2.5 Statistical analysis

Descriptive statistics were analyzed using SPSS 26.0. Normality of continuous variables was assessed with the Shapiro–Wilk test. Normally distributed data are presented as the mean (standard deviation) and were compared using independent-samples t-tests, whereas non-normally distributed data are reported as the median (interquartile range) and were compared using the Mann–Whitney U test. Categorical variables are expressed as frequencies (percentages) and were compared using the χ² test or Fisher’s exact test, as appropriate. To account for the increased risk of Type I errors due to multiple comparisons of baseline characteristics, raw values were adjusted using the Benjamini--Hochberg procedure to control the False Discovery Rate (FDR). An adjusted *p*-value (FDR q-value) < 0.05 was considered statistically significant for these comparisons. For individual statistical tests outside the baseline comparison, a standard two-sided *p*-value < 0.05 was utilized. Model performance was evaluated using the area under the receiver operating characteristic curve (AUC), sensitivity, specificity, positive predictive value (PPV), and decision curve analysis (DCA). Additionally, to assess the reliability of the predicted probabilities for clinical deployment, model calibration was evaluated using Brier scores and visualized via calibration plots. A lower Brier score indicates better agreement between predicted probabilities and observed outcomes. As a sensitivity analysis to validate the robustness of our chosen class-weighting strategy for handling the imbalanced dataset (CRPA vs. non-CRPA), we alternatively applied the Synthetic Minority Over-sampling Technique (SMOTE) strictly to the training set. The model trained on the SMOTE-balanced data was then evaluated on the untouched original test set.

## 3 Results

### 3.1 Baseline characteristics of the study cohorts

A total of 1,666 patients with *P. aeruginosa* infection were included in the development cohort, comprising 1,443 patients with non-carbapenem-resistant *P. aeruginosa* (non-CRPA) and 223 patients with carbapenem-resistant *P. aeruginosa* (CRPA). The cohort comprised 1,054 males and 612 females, aged 18–99 years. The clinical specimens were sourced primarily from the respiratory tract (sputum/bronchoalveolar lavage) (72.45%), abdominal fluid (7.44%), and urine (4.26%). Compared with the non-CRPA group, the CRPA group had a higher proportion of males (77.13% vs. 61.12%, *p* < 0.001), was older [69.15 (13.39) vs. 65.25 (19.10) years, *p* = 0.003], and had a higher ICU admission rate (30.49% vs. 7.90%, *p* < 0.001). Following Benjamini-Hochberg FDR correction, significant between-group differences remained in multiple CBC parameters: the CRPA group exhibited lower lymphocyte counts and percentages; lower erythrocyte-related indices (RBC, HGB, and HCT); higher red cell distribution width (RDW); and lower platelet indices (MPV, PDW, and PLCR) (all *p* < 0.01). Inflammatory indices, including SII, SIRI, NLR, and PLR, were also higher in the CRPA group (all *p* < 0.05) (**Table 1**). Baseline characteristics did not differ significantly between the internal and external validation cohorts (**Supplementary Table 3**).

**Table 1:** Baseline demographic characteristics, blood count parameters and derived indicators.

Variable	Non-CRPA (n=1443)	CRPA (n=223)	*p*-value	Adjusted *p*-value (FDR)
Male [n (%)]	882 (61.12%)	172 (77.13%)	<0.001	<0.001
Age (years)	65.25 (19.10)	69.15 (13.39)	0.003	0.008
WBC (×10^9^/L)	8.35 (6.01-11.73)	9.07 (6.13-11.29)	0.427	0.509
N (×10^9^/L)	6.21 (4.09-9.48)	6.69 (4.22-9.47)	0.205	0.282
N (%)	74.28 (15.09)	76.31 (14.21)	0.059	0.085
L (×10^9^/L)	1.05 (0.69-1.51)	0.96 (0.58-1.35)	0.009	0.017
L (%)	13.30 (7.45-21.50)	11.40 (6.25-17.75)	0.009	0.017
Mon (×10^9^/L)	0.59 (0.42-0.82)	0.61 (0.38-0.97)	0.449	0.511
Mon (%)	7.10 (5.50-9.30)	7.50 (5.60-9.70)	0.362	0.460
Eo (×10^9^/L)	0.07 (0.02-0.15)	0.06 (0.01-0.19)	0.863	0.863
Eo (%)	0.80 (0.20-2.00)	0.80 (0.10-2.30)	0.697	0.742
Bas (×10^9^/L)	0.03 (0.02-0.04)	0.02 (0.02-0.04)	0.057	0.085
Bas (%)	0.30 (0.20-0.50)	0.30 (0.20-0.40)	0.034	0.053
RBC (×10^12^/L)	4.06 (0.80)	3.89 (1.01)	0.004	0.009
HGB (g/L)	117.21 (22.69)	108.43 (23.47)	<0.001	<0.001
HCT (%)	36.59 (6.59)	34.05 (7.15)	<0.001	<0.001
RDW-CV (%)	14.65 (2.19)	15.45 (2.13)	<0.001	<0.001
RDW-SD (fL)	47.96 (7.09)	49.22 (6.83)	0.013	0.023
MCV (fL)	90.93 (8.49)	88.97 (8.87)	0.001	0.003
MCH (pg)	29.11 (3.21)	28.39 (3.45)	0.002	0.006
MCHC (g/L)	319.79 (16.29)	318.70 (17.71)	0.360	0.459
PLT (×10^9^/L)	214.44 (100.12)	216.77 (96.95)	0.746	0.769
MPV (fL)	10.90 (9.90-12.30)	10.40 (9.50-11.35)	<0.001	<0.001
PCT (%)	0.23 (0.18-0.30)	0.24 (0.18-0.29)	0.679	0.742
PDW (%)	12.80 (10.60-16.10)	11.70 (9.90-13.70)	<0.001	<0.001
PLCR (%)	31.80 (23.70-42.45)	27.30 (20.35-36.00)	<0.001	<0.001
LMR	1.79 (1.07-2.84)	1.50 (0.95-2.36)	<0.001	<0.001
NLR	5.72 (3.12-11.47)	6.68 (3.89-13.75)	0.017	0.028
NPR	0.03 (0.02-0.05)	0.03 (0.02-0.05)	0.432	0.509
SII	1111.00 (573.88-2440.34)	1344.20 (695.27-3410.83)	0.005	0.011
SIRI	3.26 (1.56-7.73)	4.29 (1.86-9.41)	0.013	0.023
PLR	187.04 (121.01-296.88)	224.56 (142.77-366.14)	<0.001	<0.001
Department (ICU) [n (%)]	114 (7.90%)	68 (30.49%)	<0.001	<0.001

### 3.2 Feature selection using LASSO regression

**Figure 2:**
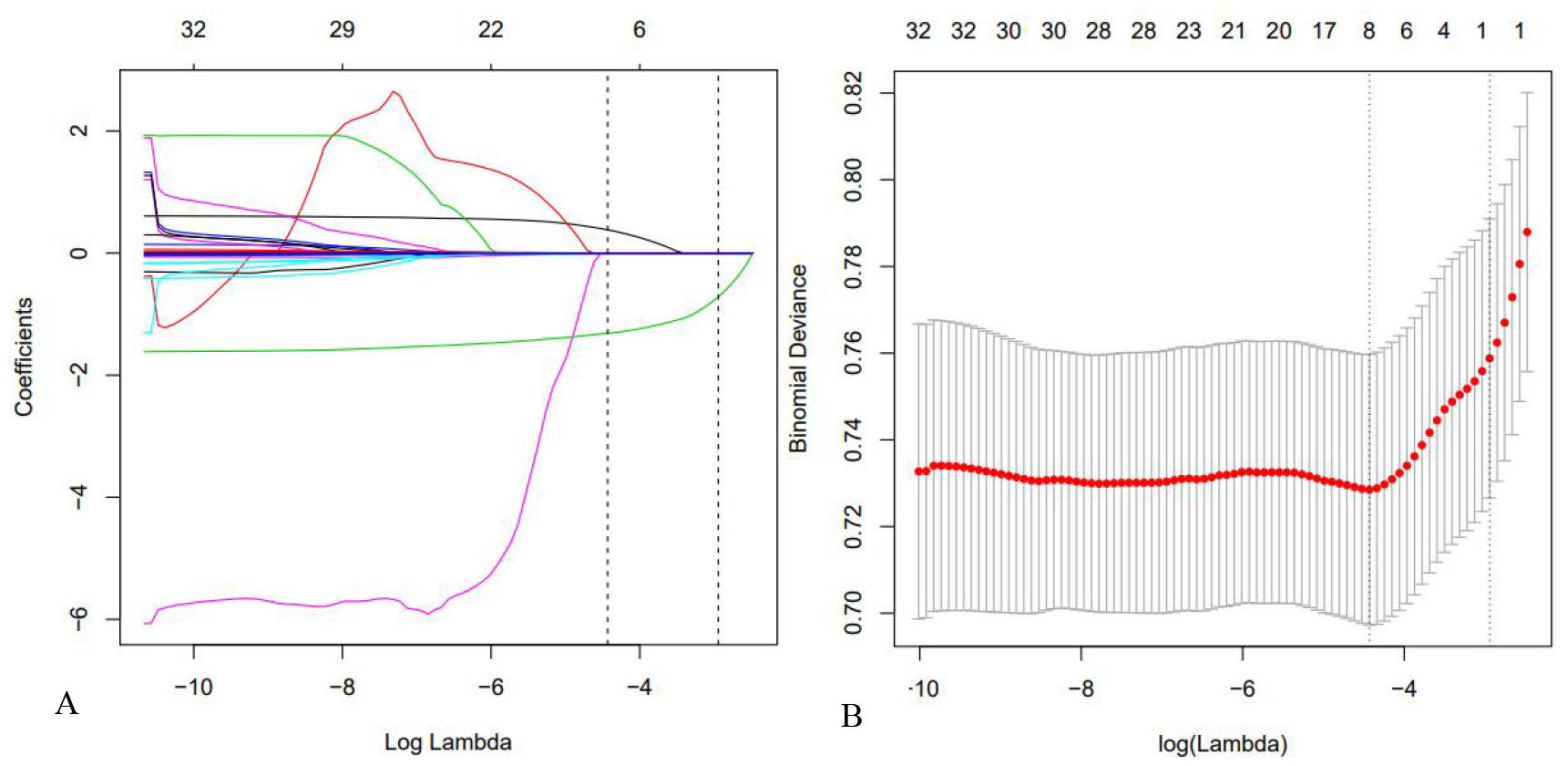
LASSO regression screening procedure. **(A)** LASSO regression cross-validation plot; **(B)** LASSO regression path plot.

To mitigate multicollinearity among variables, the least absolute shrinkage and selection operator (LASSO) regression was used for feature selection. All baseline variables were entered into the model, with categorical variables encoded as binary indicators (e.g., male = 1, female = 0; ICU = 1, non-ICU = 0). The LASSO cross-validation curve and coefficient paths are presented in **Figures 2A, B**. At the optimal lambda (λ) value, seven predictors were retained: sex, age, mean corpuscular volume (MCV), hemoglobin (HGB), platelet-to-lymphocyte ratio (PLR), systemic inflammatory response index (SIRI), and ICU admission status.

### 3.3 Model development and performance comparison

**Figure 3:**
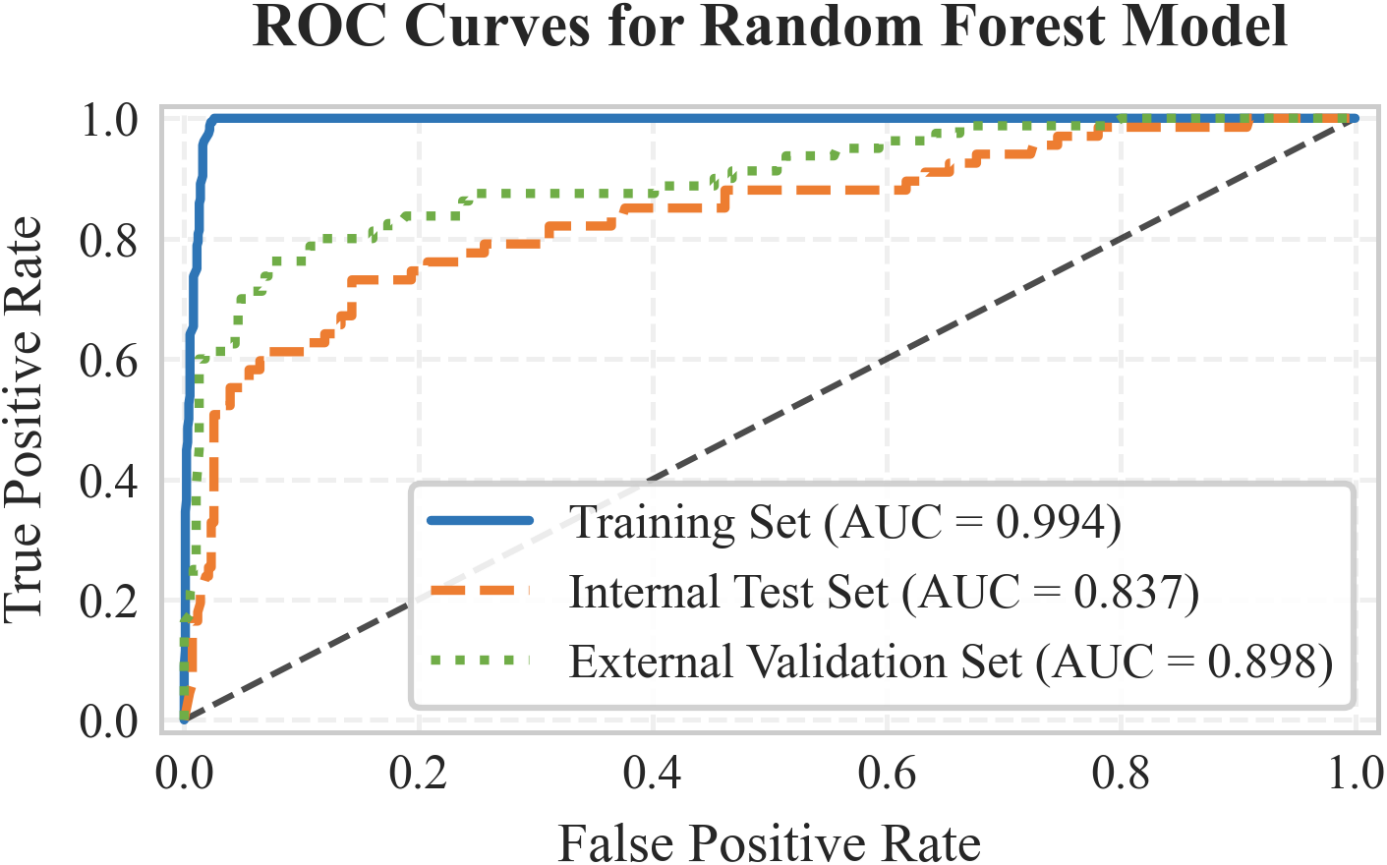
ROC curves of the random forest algorithm.

**Figure 4:**
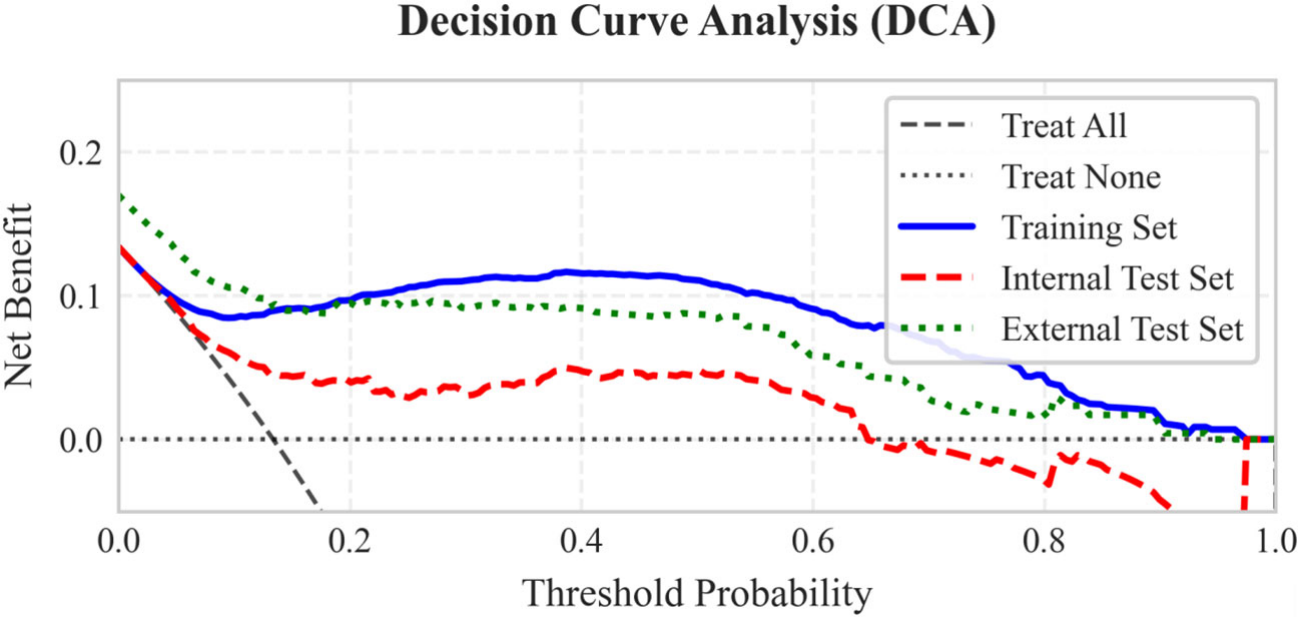
Clinical decision curve analysis based on the random forest algorithm.

**Figure 5:**
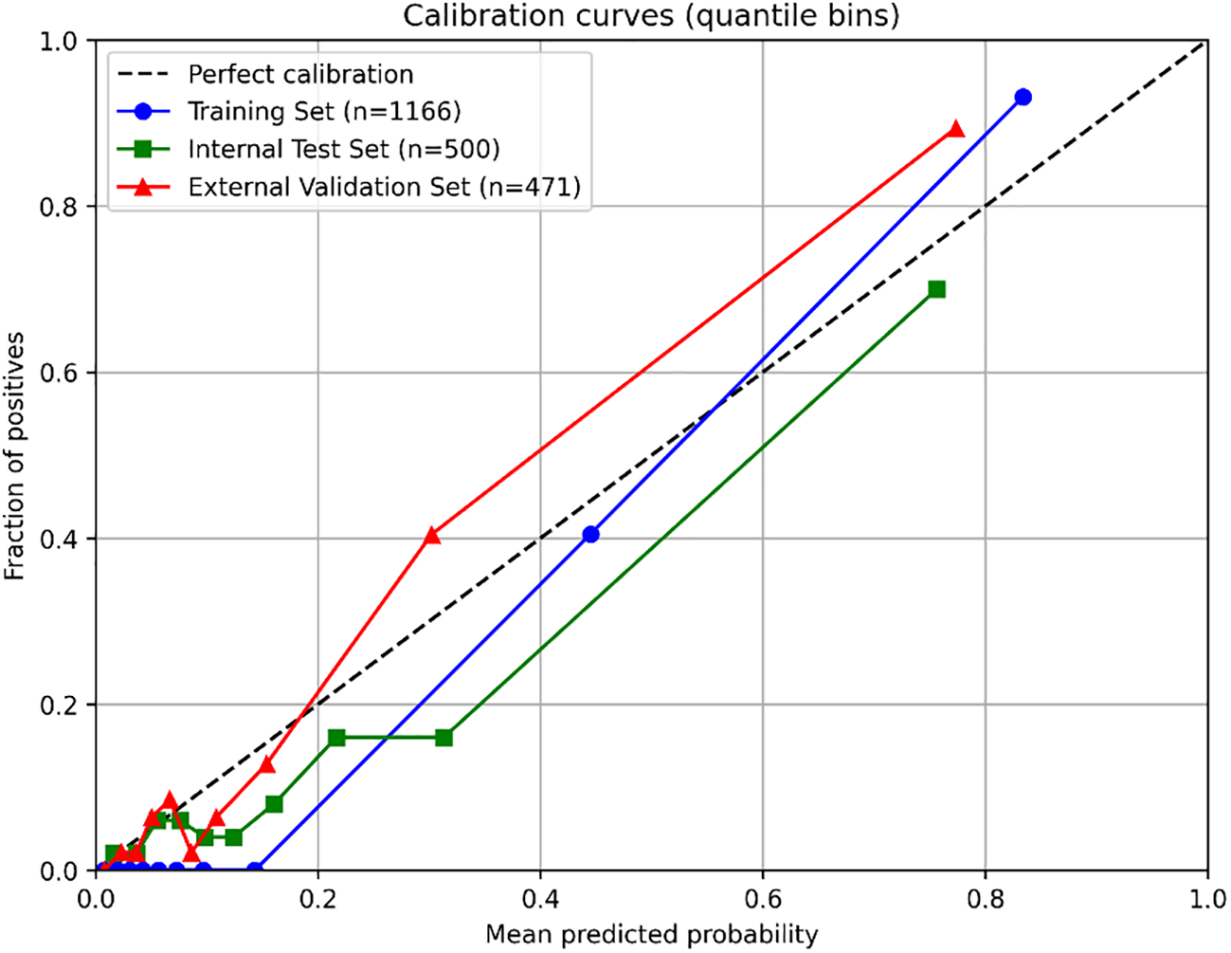
Calibration curve analysis based on the random forest algorithm.

The seven LASSO-selected features were used to train eight ML models. To comprehensively assess and mitigate the risk of overfitting inherent in complex models, 5-fold cross-validation was strictly applied. The random forest (RF) algorithm demonstrated a training AUC of 0.993, but more importantly, it achieved an average 5-fold cross-validation AUC of 0.929 ± 0.005, indicating stable discriminative capability. After hyperparameter optimization, model performance was evaluated on the internal test set (n=500). The random forest (RF) algorithm demonstrated the best overall discriminative ability, achieving an AUC of 0.837 (95% CI: 0.779–0.893). It showed very high specificity (0.972) and PPV (0.739), with a sensitivity of 0.507 (**Table 2**). Sensitivity analysis using SMOTE yielded comparable predictive performance on the internal test set (AUC 0.835 vs. 0.837) compared to the class-weighted model (**Supplementary Table 4**), confirming the robustness of our approach to handling class imbalance. The RF model maintained excellent performance in the external validation cohort (n=471), with an AUC of 0.898 (95% CI: 0.856–0.937), specificity of 0.985, PPV of 0.889, and improved sensitivity of 0.600 (**Figure 3**). Decision curve analysis (**Figure 4**) demonstrated that using the RF model for clinical decision-making provided a higher net benefit than the “treat-all” or “treat-none” strategies across a clinically relevant threshold probability range of approximately 15% to 65% in both internal and external cohorts. Furthermore, the calibration of the RF model was rigorously assessed. The model demonstrated excellent calibration in both the internal test set (Brier score: 0.084) and the external validation cohort (Brier score: 0.073). The calibration plots (**Figure 5**) showed a high degree of agreement between the predicted probabilities of CRPA infection and the actual observed frequencies, reinforcing the model’s reliability for clinical deployment.

### 3.4 Model interpretability and clinical application

**Figure 6:**
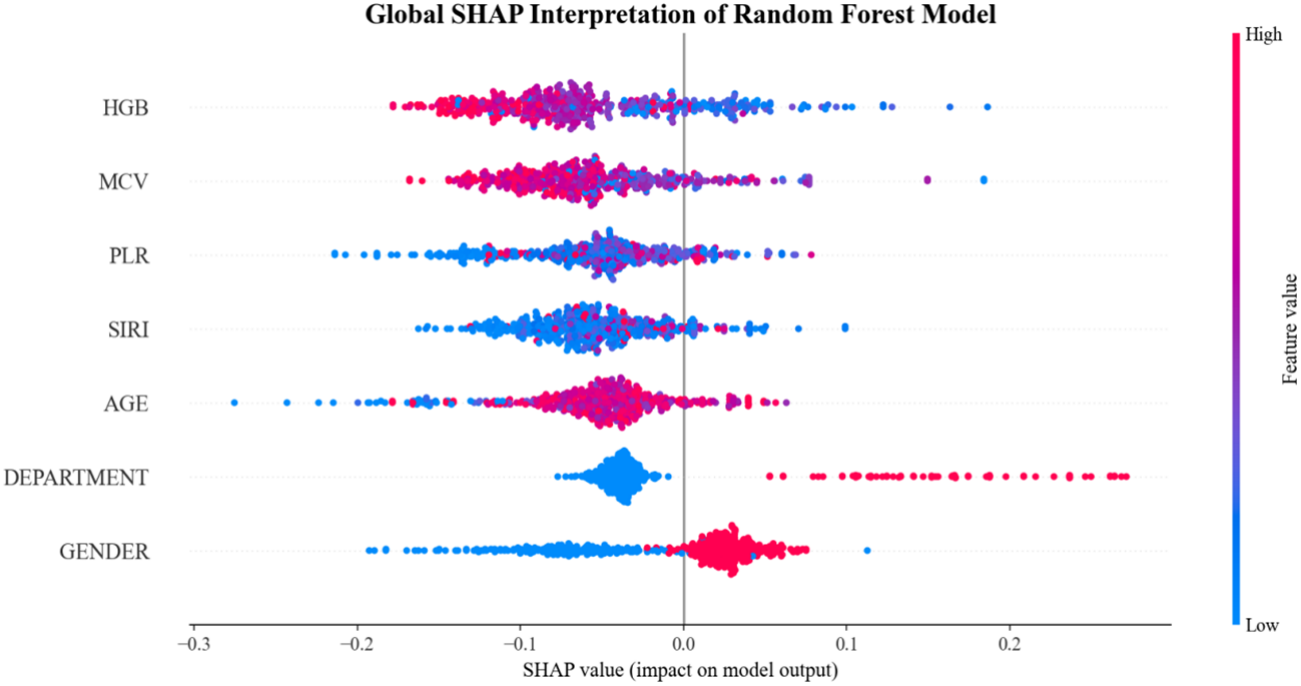
Interpretability of the random forest model using SHAP values.

**Figure 7:**
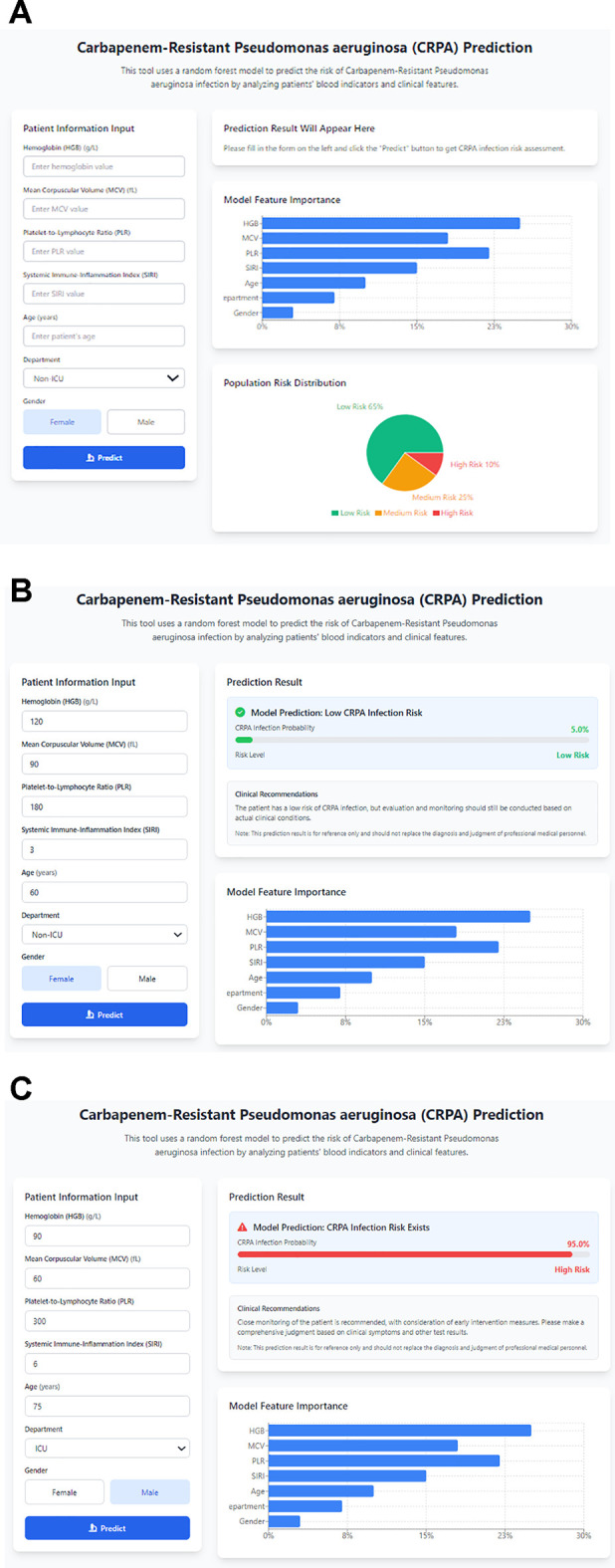
Web-based calculator interface and examples for CRPA infection risk prediction based on seven clinical and laboratory features. **(A)** Sample interface of the web calculator, showing input fields (hemoglobin, MCV, PLR, SIRI, age, sex, ICU admission status), a bar chart of model feature importance, and a pie chart of population risk distribution. **(B)** Low-risk example: a 60-year-old female non-ICU patient with a predicted CRPA risk of 5.0%. **(C)** High-risk example: a 75-year-old male ICU patient with a predicted CRPA risk of 95.0%.

To interpret the RF model, Shapley Additive Explanations (SHAP) analysis was conducted. The global SHAP summary plot (**Figure 6**) indicated that hemoglobin concentration was the most influential predictor and was inversely associated with the risk of CRPA infection. To facilitate clinical implementation, a web-based calculator incorporating the seven predictive features was developed (**Figure 7A**). This tool enables clinicians to enter patient parameters and obtain an estimated probability of CRPA infection. Two illustrative cases are provided: a 60-year-old female non-ICU patient with a low predicted risk (5.0%) and a 75-year-old male ICU patient with a high predicted risk (95.0%), demonstrating the potential utility of the tool in informing infection-control and treatment decisions (**Figures 7B, C**). The calculator is publicly available at https://space.coze.cn/coding-expert-runtime/328217891330?task_id=7596485525121777962.

**Table 2:** Comparison of model performance.

Model	Sensitivity	Specificity	Accuracy	AUC (95% CI)	AUCPR (95% CI)	PPV	NPV	Balanced accuracy	F1- score	Brier score
Training										
RF	0.994	0.977	0.979	0.993 (0.810-1.000)	0.947 (0.827-0.957)	0.871	0.999	0.985	0.928	0.025
LG	0.577	0.706	0.689	0.718 (0.668-0.730)	0.294 (0.279-0.305)	0.233	0.915	0.641	0.331	0.210
DT	0.981	0.825	0.846	0.783 (0.738-0.850)	0.400 (0.317-0.540)	0.464	0.996	0.903	0.630	0.095
SVM	0.346	0.884	0.812	0.718 (0.697-0.739)	0.297 (0.247-0.347)	0.316	0.897	0.615	0.330	0.106
KNN	0.904	0.995	0.983	0.999 (0.997-1.000)	0.989 (0.939-1.000)	0.966	0.985	0.949	0.934	0.010
GBM	0.917	0.993	0.983	0.999 (0.997-1.000)	0.990 (0.940-1.000)	0.953	0.987	0.955	0.935	0.011
XGB	0.244	0.995	0.895	0.868 (0.853-0.883)	0.626 (0.576-0.676)	0.884	0.895	0.619	0.382	0.081
LGBM	0.910	0.994	0.983	0.998 (0.997-1.000)	0.988 (0.938-1.000)	0.959	0.986	0.952	0.934	0.014
Internal test										
RF	0.507	0.972	0.910	0.837 (0.779-0.893)	0.535 (0.418-0.674)	0.739	0.927	0.740	0.602	0.084
LG	0.657	0.686	0.682	0.720 (0.650-0.784)	0.289 (0.215-0.400)	0.244	0.928	0.671	0.356	0.219
DT	0.672	0.725	0.718	0.754 (0.692-0.814)	0.374 (0.263-0.496)	0.274	0.935	0.698	0.390	0.178
SVM	0.433	0.868	0.810	0.722 (0.654-0.793)	0.303 (0.225-0.408)	0.337	0.908	0.651	0.379	0.106
KNN	0.403	0.975	0.898	0.818 (0.756-0.881)	0.541 (0.430-0.661)	0.711	0.913	0.689	0.514	0.087
GBM	0.418	0.965	0.892	0.814 (0.764-0.876)	0.483 (0.372-0.615)	0.651	0.915	0.692	0.509	0.093
XGB	0.209	0.984	0.880	0.762 (0.703-0.828)	0.421 (0.326-0.527)	0.667	0.889	0.596	0.318	0.099
LGBM	0.388	0.954	0.878	0.830 (0.777-0.888)	0.500 (0.397-0.625)	0.565	0.910	0.671	0.460	0.094
External test										
RF	0.600	0.985	0.919	0.898 (0.856-0.937)	0.757 (0.666-0.843)	0.889	0.923	0.792	0.716	0.073
LG	0.550	0.729	0.699	0.698 (0.639-0.758)	0.312 (0.239-0.420)	0.293	0.888	0.639	0.383	0.212
DT	0.688	0.852	0.824	0.807 (0.748-0.866)	0.523 (0.416-0.643)	0.200	0.847	0.539	0.272	0.123
SVM	0.250	0.882	0.775	0.705 (0.650-0.769)	0.323 (0.248-0.439)	0.303	0.852	0.566	0.274	0.135
KNN	0.537	0.990	0.913	0.935 (0.905-0.962)	0.812 (0.730-0.876)	0.915	0.913	0.764	0.677	0.065
GBM	0.525	0.987	0.909	0.528 (0.519-0.651)	0.228 (0.177-0.338)	0.894	0.910	0.756	0.661	0.075
XGB	0.138	0.997	0.851	0.783 (0.730-0.840)	0.533 (0.439-0.634)	0.917	0.850	0.567	0.239	0.114
LGBM	0.525	0.985	0.907	0.843 (0.788-0.900)	0.749 (0.670-0.826)	0.875	0.910	0.755	0.656	0.075

RF, random forest; LG, logistic regression; DT, decision trees; SVM, support vector machines; KNN, k-nearest neighbor algorithm; GBM, gradient boosting machines; XGB, eXtreme gradient boosting; LGBM, Light gradient boosting machine; AUC, area under the receiver operating characteristic curve; AUCPR, area under the precision-recall curve; PPV, positive predictive value; NPV, negative predictive value.

## 4 Discussion

Carbapenem-resistant *Pseudomonas aeruginosa* (CRPA) infections pose a major therapeutic challenge, which is further exacerbated by the diagnostic delays associated with conventional culture-based methods (Tamma et al., 2023). Accordingly, rapid and accessible tools are needed to support early empirical therapy. Machine-learning (ML) approaches applied to electronic health records provide a promising strategy for predicting antimicrobial resistance (İlhanlı et al., 2024; Blechman and Wright, 2024; Cui et al., 2025). In this study, we developed and validated an interpretable, clinlabomic machine-learning model for early risk stratification of carbapenem resistance among confirmed *P. aeruginosa* infected patients using only routine CBC parameters and basic demographics. Our findings are consistent with the growing emphasis on leveraging widely available clinical data for antimicrobial-resistance surveillance (Boolchandani et al., 2019).

As demonstrated in our results, while our random forest model demonstrated robust discriminatory power (AUCs: 0.837–0.898), its performance profile is characterized by modest sensitivity (0.507–0.600) and exceptionally high specificity (>0.97), consistent with similar high-specificity screening tools (Blechman and Wright, 2024). Consequently, this model should be positioned as a “rule-in” diagnostic adjunct rather than a broad screening instrument. The clinical implications of this performance profile warrant careful consideration. Given the modest sensitivity, the model will inherently miss approximately 40-50% of true CRPA cases (false negatives). Therefore, a low predicted probability should not be used to prematurely withhold or de-escalate broad-spectrum empiric therapy if a patient is critically ill or clinical suspicion remains high. Instead, the true clinical utility of our model lies in its remarkable specificity and high positive predictive value (PPV). When the model flags a patient as high-risk, clinicians can be highly confident in the prediction. In the critical 48–72 hours before culture results are finalized, a positive prediction could prompt the immediate initiation of targeted anti-CRPA therapies (e.g., novel β-lactam/β-lactamase inhibitor combinations) and the prompt implementation of strict contact precautions and infection control measures. Furthermore, to fulfill the prerequisites for clinical deployment, our model demonstrated strong calibration (Brier scores: 0.073–0.084), ensuring that the risk probabilities provided by our web-based calculator accurately reflect the real-world likelihood of CRPA infection.

While severe infections frequently trigger inflammation-induced hypoferremia and anemia, it is crucial to emphasize that our model identifies statistical associations rather than mechanistic pathways. Although it is biologically plausible that anemia reflects an altered host microenvironment or a state of chronic debilitation conducive to multidrug-resistant colonization, the current data cannot support direct causal inferences. Further prospective mechanistic studies are required to elucidate the exact relationship between host iron metabolism and CRPA pathophysiology.

We observed a substantial performance gap in the random forest model between the training set (AUC 0.993) and the internal test set (AUC 0.837). Addressing this discrepancy is critical for validating the model’s clinical reliability. First, we rigorously ruled out “data leakage” as a cause; temporal and stratified data splitting, along with standard scaling (Z-score normalization), were strictly performed after the dataset partition, ensuring no future or test-set information contaminated the training phase. Therefore, this gap is primarily attributable to the inherent “model complexity” of the random forest algorithm interacting with “data noise.” Random forest is an ensemble of deep decision trees highly prone to memorizing the idiosyncratic noise and minor fluctuations inherent in retrospective, real-world clinical laboratory data (e.g., patient-to-patient biological variance in CBC parameters) during the training phase, leading to near-perfect training metrics. However, rather than viewing this purely as detrimental overfitting, we emphasize that the 5-fold cross-validated mean AUC (0.929) and the robust performance on the unseen internal test set (AUC 0.837) and independent external cohort (AUC 0.898) confirm that the model successfully extracted underlying, generalizable biological patterns despite memorizing some training noise. Despite the overfitting on the training set, the robust test performance indicates that the clinlabomic signatures captured by the model remain clinically viable across different patient populations.

Previous efforts to predict antimicrobial resistance have leveraged diverse data sources. Models based on whole-genome sequencing (WGS) can achieve high accuracy but are constrained by cost, technical complexity, and turnaround time, limiting their utility for rapid bedside screening (Liu et al., 2023). Other models incorporating clinical risk factors, such as ICU admission and prior antibiotic exposure, have also shown promise (Jiang et al., 2025). In contrast, our model relies solely on routinely available CBC parameters, offering a practical trade-off between predictive performance (AUC: 0.837–0.898) and clinical feasibility for early point-of-care risk stratification.

The seven predictors identified by LASSO regression—sex, age, mean corpuscular volume (MCV), hemoglobin (HGB), platelet-to-lymphocyte ratio (PLR), systemic inflammatory response index (SIRI), and ICU admission status—collectively define a high-risk profile for CRPA infection. Advanced age and ICU exposure are well-established risk factors for multidrug-resistant infections (Martin-Mateos et al., 2024; Liu et al., 2024). Notably, SHAP analysis identified hemoglobin (HGB) as a paramount predictor. While severe infections frequently trigger inflammation-induced hypoferremia and anemia (Ganz, 2019), it is crucial to emphasize that our model identifies statistical associations rather than mechanistic pathways. Although it is biologically plausible that anemia reflects an altered host microenvironment or a state of chronic debilitation conducive to multidrug-resistant colonization, the current data cannot support direct causal inferences. Further prospective mechanistic studies are required to elucidate the exact relationship between host iron metabolism and CRPA pathophysiology. Importantly, although hemoglobin contributed substantially to model predictions, conventional markers of acute infection, such as white blood cell count, did not differ significantly between groups, suggesting that resistance prediction may depend on subtler, integrated clinlabomics signatures rather than overt inflammatory changes (Lundberg et al., 2018).

The predictive value of derived inflammatory indices, particularly SIRI and PLR, further underscores an altered host immune state in CRPA infection. Their elevation suggests systemic inflammation accompanied by relative immune dysregulation (Kimak-Pielas et al., 2025). These indices have been associated with mortality in sepsis and severe infections (Mangalesh et al., 2023; Kollu et al., 2025), consistent with the high attributable mortality reported for CRPA bloodstream infections (Falcone et al., 2023). Thus, our model integrates demographic, hematological, and inflammatory signals to characterize a host phenotype featuring advanced age, critical illness exposure, reduced physiological reserve, and systemic inflammation—features that are consistent with increased susceptibility to CRPA.

The association between these clinlabomics features and CRPA risk may reflect a dysregulated systemic inflammatory response, as suggested by elevated SIRI and PLR. Elevated PLR and SIRI reflect a pro-inflammatory milieu that may impair local immune surveillance and modify the host microenvironment, potentially facilitating colonization and infection by resistant pathogens such as *P. aeruginosa* (Pang et al., 2019; Alparslan et al., 2025). In this context, the model may capture a pre-existing inflammatory phenotype that predisposes individuals to CRPA, providing a biological rationale for the predictive value of routine laboratory parameters.

Beyond the host immune-inflammatory response, the specific microbiological features of CRPA infections warrant careful consideration to fully contextualize our model’s predictions. Regarding virulence, it is a complex paradigm; while CRPA isolates are not inherently more virulent than carbapenem-susceptible strains—and may even suffer a biological “fitness cost” due to the burden of maintaining resistance mechanisms—they frequently belong to epidemic high-risk global clones (e.g., ST235) that uniquely combine multidrug resistance with enhanced virulence traits, such as the type III secretion system and ExoU toxin (Horcajada et al., 2019).

Furthermore, the predicted resistance mechanisms of CRPA are highly relevant to the clinical phenotype identified by our model. Carbapenem resistance in *P. aeruginosa* is extraordinarily complex, driven by both mutational (chromosomally encoded) and horizontally acquired mechanisms. The most prevalent mechanism globally is the mutational inactivation or downregulation of the chromosomally encoded OprD porin (restricting imipenem entry), frequently coupled with the overexpression of chromosomal efflux pumps like MexAB-OprM (extruding meropenem) and the derepression of AmpC β-lactamase (Pang et al., 2019). These chromosomal mutations are typically selected sequentially under sustained antibiotic pressure during prolonged hospitalizations. Less frequently but increasingly alarming is the acquisition of mobile genetic elements (plasmids or integrons) encoding exogenous carbapenemases (e.g., metallo-β-lactamases such as VIM, IMP, NDM, or serine carbapenemases like KPC) (Yoon and Jeong, 2021).

From a clinlabomic perspective, patients harboring CRPA with these mechanisms typically represent a highly debilitated population. The chronic antibiotic exposure required to select for chromosomal OprD loss or the acquisition of plasmid-mediated carbapenemases often coincides with prolonged ICU stays, recurrent infections, and a state of persistent immune exhaustion. This clinical trajectory perfectly mirrors the host phenotype captured by our machine-learning model: advanced age, profound systemic inflammation (elevated SIRI and PLR), and significant metabolic/nutritional depletion (low HGB and MCV). Thus, our model likely acts as a surrogate detector for this intersection: a vulnerable, inflamed host microenvironment colonized by a pathogen equipped with complex, high-burden resistance mechanisms.

This study has several limitations. First, the retrospective design may introduce selection bias. Second, to prioritize model parsimony and facilitate rapid, automated risk assessment using readily available laboratory parameters, we intentionally excluded several classical and critical clinical risk factors for CRPA, including prior exposure to broad-spectrum antibiotics, history of prolonged hospitalization, specific chronic comorbidities, and recent invasive procedures (e.g., mechanical ventilation). While this design allows the model to function without requiring time-consuming manual chart reviews, we acknowledge that this omission significantly limits the comprehensive clinical validity of the model. Third, our cohort included varying infection sites (predominantly respiratory, but also abdominal and urinary tracts). The clinical heterogeneity of these infections, alongside the potential presence of unrecorded polymicrobial infections, introduces inherent confounders. Furthermore, our study did not distinguish between hospital-acquired and community-acquired infections. Given that these two entities differ significantly in their clinical significance and baseline resistance probabilities, the lack of this distinction, alongside the potential presence of unrecorded polymicrobial infections, introduces inherent confounders. Consequently, it is possible that our model partially captures a proxy signature for overall illness severity and severe host debilitation rather than a phenotype exclusively specific to CRPA.

Despite these limitations, the clinical implications are significant. In the critical hours before AST results, our model can provide a data-driven probability of CRPA. As a clinical tool, our model exhibits extremely high specificity but modest sensitivity. This asymmetrical performance profile dictates its use as a strict “rule-in” adjunct. Clinicians can leverage a positive prediction to confidently initiate early targeted anti-CRPA therapy and enforce isolation precautions. However, the model will inevitably miss a proportion of true CRPA cases. Therefore, a negative prediction should never be used in isolation to safely withhold or de-escalate empirical broad-spectrum antibiotics. This aligns precisely with antimicrobial stewardship goals: optimizing therapy for the individual while reducing unnecessary selection pressure. The deployed web calculator lowers the barrier to implementation for real-time clinical decision support. .

## 5 Conclusion

In conclusion, we have developed and validated a parsimonious, interpretable machine learning model that leverages routine clinlabomic data to stratify the early risk of carbapenem-resistant *Pseudomonas aeruginosa* among infected patients with high specificity. By identifying a signature of anemia, systemic inflammation, and critical illness, the model provides a biological lens into the host environment associated with CRPA. Functioning as a robust “rule-in” tool, it offers a rapid, low-cost strategy to inform the early escalation of empirical antibiotic choices and support antimicrobial stewardship efforts. Future work should focus on prospective validation in diverse settings, incorporating prior antibiotic histories, and integration into clinical workflow systems.

## Data Availability

The raw data supporting the conclusions of this article will be made available by the authors, without undue reservation.
