# Supplementary material for: Early risk stratification for carbapenem resistance among Pseudomonas aeruginosa infected patients using a clinico-laboratory machine-learning model based on routine complete blood count parameters
Source: Front Cell Infect Microbiol. 2026 Apr 16;16:1795720. doi: 10.3389/fcimb.2026.1795720 (PMC13128656; doi:10.3389/fcimb.2026.1795720)
Supplement: Supplementary file 1 [file Table1.docx]

Supplementary Material

**Supplementary Table 1** Baseline characteristics comparison between training and internal test sets

| Variable | Training set (n=1116) | Internal test set (n=500) | *p*-value |
| --- | --- | --- | --- |
| Male [n (%)] | 733 (62.9%) | 733 (62.9%) | 0.644 |
| Age (years) | 65.93 (18.28) | 65.37 (18.95) | 0.569 |
| WBC (×10^9^/L) | 8.38 (5.93-11.50) | 8.83 (6.12-12.09) | 0.160 |
| N (×10^9^/L) | 6.26 (4.04-9.28) | 6.38 (4.13-9.99) | 0.212 |
| N (%) | 74.91 (14.53) | 73.69 (15.98) | 0.144 |
| L (×10^9^/L) | 1.05 (0.70-1.51) | 1.02 (0.63-1.47) | 0.216 |
| L (%) | 13.40 (7.30-21.30) | 12.40 (6.78-19.82) | 0.096 |
| Mon (×10^9^/L) | 0.58 (0.42-0.82) | 0.60 (0.42-0.84) | 0.637 |
| Mon (%) | 7.20 (5.60-9.40) | 7.15 (5.50-9.30) | 0.475 |
| Eo (×10^9^/L) | 0.07 (0.02-0.15) | 0.06 (0.01-0.14) | 0.200 |
| Eo (%) | 0.90 (0.20-2.10) | 0.70 (0.10-1.90) | 0.132 |
| Bas (×10^9^/L) | 0.03 (0.02-0.04 | 0.02 (0.01-0.04) | 0.091 |
| Bas (%) | 0.30 (0.20-0.50) | 0.30 (0.20-0.40) | 0.084 |
| RBC (×10^12^/L) | 4.03 (0.80) | 4.04 (0.90) | 0.849 |
| HGB (g/L) | 116.16 (23.11) | 115.73 (22.69) | 0.726 |
| HCT (%) | 36.31 (6.69) | 36.11 (6.79) | 0.585 |
| RDW-CV (%) | 14.80 (3.07) | 14.82 (2.29) | 0.925 |
| RDW-SD (fL) | 48.40 (9.50) | 48.07 (7.79) | 0.503 |
| MCV (fL) | 90.74 (8.25) | 90.52 (9.27) | 0.650 |
| MCH (pg) | 29.01 (3.21) | 29.01 (3.37) | 0.966 |
| MCHC (g/L) | 319.41 (16.81) | 320.20 (15.70) | 0.365 |
| PLT (×10^9^/L) | 214.36 (95.05) | 215.67 (109.80) | 0.816 |
| MPV (fL) | 10.80 (9.80-12.20) | 10.80 (9.90-12.20) | 0.741 |
| PCT (%) | 0.23 (0.18-0.30) | 0.23 (0.18-0.30) | 0.199 |
| PDW (%) | 12.55 (10.40-16.10) | 12.70 (10.60-15.80) | 0.772 |
| PLCR (%) | 31.05 (23.10-42.27) | 31.45 (24.00-41.70) | 0.717 |
| LMR | 1.77 (1.05-2.78) | 1.64 (1.02-2.69) | 0.118 |
| NLR | 5.63 (3.12-11.59) | 6.20 (3.42-12.71) | 0.078 |
| NPR | 0.03 (0.02-0.05) | 0.03 (0.02-0.05) | 0.073 |
| SII | 1127.65 (578.14-2516.38) | 1146.26 (603.42-2638.34) | 0.301 |
| SIRI | 3.30 (1.55-7.71) | 3.89 (1.68-8.74) | 0.108 |
| PLR | 188.28 (124.09-303.69) | 192.49 (122.66-296.63) | 0.894 |
| Department (ICU) [n (%)] | 117 (10.03%) | 65 (13.00%) | 0.091 |
| CRPA [n (%)] | 156 (13.38%) | 67 (13.40%) | 1.000 |

**Supplementary Table 2** Hyperparameters of machine learning models

| **Model** | **Hyperparameter** | **Range** | **Result** |
| --- | --- | --- | --- |
| Random forest | n_estimators | [10, 500] | 206 |
|  | max_depth | [2, 20] | 13 |
|  | min_samples_split | [2, 20] | 2 |
|  | min_samples_leaf | [1, 10] | 1 |
|  | max_features | [0.1, 1.0] | 0.1 |
| Logistic regression | C | [0.001, 10] | 1.801 |
|  | penalty | {l1, l2, elasticnet} | elastic network (L1 ratio = 0.631) |
|  | l1_ratio | [0, 1] | 0.631 |
|  | solver | {lbfgs, liblinear, saga} | saga |
| Decision trees | max_depth | [2, 30] | 30 |
|  | min_samples_split | [2, 50] | 16 |
|  | min_samples_leaf | [1, 20] | 4 |
|  | max_features | [0.1, 1.0] | 1.0 |
|  | min_impurity_decrease | [0.0, 0.1] | 0.0 |
| Support vector machines | C | [0.001, 2] | 0.001 |
|  | gamma | [0.0001, 1] | 0.000 |
|  | kernel_type | {linear, rbf } | linear |
| K-nearest neighbour algorithm | n_neighbors | [1, 50] | 22 |
|  | weights | {uniform, distance} | distance |
|  | p | [1, 5] | 3.95 |
|  | algorithm | {auto, ball_tree, kd_tree, brute} | brute |
| Gradient boosting machines | n_estimators | [10, 200] | 82 |
|  | learning_rate | [0.001, 0.3] | 0.300 |
|  | max_depth | [1, 8] | 8 |
|  | min_samples_split | [2, 30] | 30 |
|  | min_samples_leaf | [1, 20] | 20 |
|  | subsample | [0.5, 1.0] | 1.00 |
| eXtreme gradient boosting | n_estimators | [10, 200] | 94 |
|  | learning_rate | [0.001, 0.3] | 0.297 |
|  | max_depth | [1, 8] | 4 |
|  | min_child_weight | [1, 10] | 9.1 |
|  | subsample | [0.5, 1.0] | 0.72 |
|  | colsample_bytree \| | [0.5, 1.0] | 0.95 |
|  | gamma | [0, 5] | 1.03 |
|  | reg_alpha | [0, 5] | 2.49 |
|  | reg_lambda | [0.1, 5] | 2.49 |
| Light gradient boosting machine | n_estimators | [10, 200] | 156 |
|  | learning_rate | [0.001, 0.3] | 0.300 |
|  | max_depth | [1, 8] | 8 |
|  | num_leaves | [2, 64] | 64 |
|  | min_child_samples | [1, 50] | 50 |
|  | subsample | [0.5, 1.0] | 0.5 |
|  | colsample_bytree | [0.5, 1.0] | 1.0 |
|  | reg_alpha | [0, 5] | 0 |
|  | reg_lambda | [0, 5] | 5.0 |

**Supplementary Table 3** Baseline characteristics between the internal and external cohorts

| **Variable** | **The internal cohorts (n=1666)** | **The external cohort (n=471)** | ***P*-value** |
| --- | --- | --- | --- |
| Male [n (%)] | 766 (65.69%) | 288 (57.60%) | 0.002 |
| Age (years) | 65.60 (19.00) | 66.16 (17.23) | 0.571 |
| WBC (×10^9^/L) | 8.52 (5.91-11.90) | 8.36 (6.31-11.36) | 0.758 |
| N (×10^9^/L) | 6.34 (4.02-9.66) | 6.25 (4.33-9.23) | 0.518 |
| N (%) | 74.23 (15.23) | 75.28 (14.37) | 0.193 |
| L (×10^9^/L) | 1.04 (0.66-1.49) | 1.04 (0.72-1.54) | 0.440 |
| L (%) | 13.25 (7.00-21.28) | 13.25 (7.00-21.28) | 0.544 |
| Mon (×10^9^/L) | 0.60 (0.42-0.85) | 0.60 (0.42-0.85) | 0.076 |
| Mon (%) | 7.30 (5.60-9.47) | 7.10 (5.40-9.00) | 0.033 |
| EO (×10^9^/L) | 0.07 (0.01-0.15) | 0.06 (0.02-0.15) | 0.953 |
| EO (%) | 0.80 (0.20-2.10) | 0.90 (0.20-1.90) | 0.952 |
| BAS (×10^9^/L) | 0.03 (0.02-0.04) | 0.02 (0.02-0.04) | 0.242 |
| BAS (%) | 0.30 (0.20-0.50) | 0.30 (0.20-0.50) | 0.260 |
| RBC (×10^12^/L) | 4.05 (0.86) | 3.99 (0.78) | 0.124 |
| HGB (g/L) | 116.74 (23.05) | 114.38 (22.76) | 0.055 |
| HCT (%) | 36.31 (6.73) | 36.11 (6.72) | 0.578 |
| RDW_CV (%) | 14.74 (2.22) | 14.97 (3.97) | 0.119 |
| RDW_SD (fL) | 47.92 (7.26) | 49.18 (12.14) | 0.009 |
| MCV (fL) | 90.47 (8.74) | 91.15 (8.14) | 0.133 |
| MCH (pg) | 29.07 (3.30) | 28.86 (3.14) | 0.226 |
| MCHC (g/L) | 321.09 (16.39) | 316.27 (16.25) | <0.001 |
| PLT (×10^9^/L) | 212.57 (99.76) | 219.83 (99.40) | 0.173 |
| MPV (fL) | 10.90 (9.80-12.20) | 10.70 (9.80-12.12) | 0.918 |
| PCT (%) | 0.23 (0.18-0.30) | 0.24 (0.19-0.30) | 0.049 |
| PDW (%) | 12.70 (10.50-15.90) | 12.40 (10.49-16.10) | 0.773 |
| PLCR (%) | 31.65 (23.50-41.80) | 30.90 (23.25-42.15) | 0.953 |
| LMR | 1.68 (1.03-2.75) | 1.81 (1.17-2.77) | 0.139 |
| NLR | 5.83 (3.12-12.30) | 5.81 (3.32-11.23) | 0.602 |
| NPR | 0.03 (0.02-0.05) | 0.03 (0.02-0.05) | 0.907 |
| SII | 1123.28 (545.06-2525.62) | 1137.38 (650.74-2541.30) | 0.244 |
| SIRI | 3.42 (1.55-8.33) | 3.41 (1.64-7.54) | 0.917 |
| PLR | 188.28 (122.81-303.64) | 190.75 (124.14-298.68) | 0.743 |
| Department (ICU) [n (%)] | 120 (10.29%) | 62 (12.40%) | 0.206 |
| CRPA [n (%)] | 158 (13.55%) | 65 (13.00%) | 0.762 |

**Supplementary Table 4** Comparison of predictive performance on the untouched test set between models handling class imbalance via Class-weighting vs. SMOTE.

| Model (Strategy) | Sensitivity | Specificity | Accuracy | AUC (95% CI) | AUCPR (95% CI) | PPV | NPV | Balanced  accuracy | F1-  Score | Brier Score |
| --- | --- | --- | --- | --- | --- | --- | --- | --- | --- | --- |
| RF  (Class-weighting) | 0.507 | 0.972 | 0.910 | 0.837 (0.779-0.893) | 0.535 (0.418-0.674) | 0.739 | 0.927 | 0.740 | 0.602 | 0.084 |
| RF ( SMOTE) | 0.582 | 0.889 | 0.848 | 0.835 (0.778-0.888) | 0.507 (0.397-0.646) | 0.448 | 0.932 | 0.736 | 0.506 |  |

RF, random forest;  SMOTE, synthetic minority over-sampling technique.
